# Droplet Digital PCR for Non-Invasive Prenatal Detection of Fetal Single-Gene Point Mutations in Maternal Plasma

**DOI:** 10.3390/ijms23052819

**Published:** 2022-03-04

**Authors:** Elisabetta D’Aversa, Giulia Breveglieri, Effrossyni Boutou, Angeliki Balassopoulou, Ersi Voskaridou, Patrizia Pellegatti, Giovanni Guerra, Chiara Scapoli, Roberto Gambari, Monica Borgatti

**Affiliations:** 1 Department of Life Sciences and Biotechnology, University of Ferrara, 44121 Ferrara, Italy; elisabetta.daversa@unife.it (E.D.); giulia.breveglieri@unife.it (G.B.); chiara.scapoli@unife.it (C.S.); roberto.gambari@unife.it (R.G.); 2 Molecular Genetics Laboratory, Thalassemia and Hemoglobinopathies Center, Laiko General Hospital, 11526 Athens, Greece; e.boutou@laiko.gr (E.B.); angbalip@gmail.com (A.B.); 3 Thalassemia and Hemoglobinopathies Center, Laiko General Hospital, 11526 Athens, Greece; ersi.voskaridou@gmail.com; 4 Operative Unit of Laboratory Analysis, University Hospital S. Anna, 44121 Ferrara, Italy; p.pellegatti@ospfe.it (P.P.); gguerra@asst-pg23.it (G.G.); 5 Thal-LAB, Research Laboratory “Elio Zago” on the Pharmacologic and Pharmacogenomic Therapy of Thalassemia, University of Ferrara, 44121 Ferrara, Italy; 6 Biotechnology Center, University of Ferrara, 44121 Ferrara, Italy

**Keywords:** single point mutation, non-invasive prenatal detection, droplet digital PCR, circulating cell-free fetal DNA, maternally inherited mutations, β thalassemia

## Abstract

Non-invasive prenatal testing (NIPT) is based on the detection and characterization of circulating cell-free fetal DNA (ccffDNA) in maternal plasma and aims to identify genetic abnormalities. At present, commercial NIPT kits can detect only aneuploidies, small deletions and insertions and some paternally inherited single-gene point mutations causing genetic diseases, but not maternally inherited ones. In this work, we have developed two NIPT assays, based on the innovative and sensitive droplet digital PCR (ddPCR) technology, to identify the two most common β thalassemia mutations in the Mediterranean area (β^+^IVSI-110 and β^0^39), maternally and/or paternally inherited, by fetal genotyping. The assays were optimized in terms of amplification efficiency and hybridization specificity, using mixtures of two genomic DNAs with different genotypes and percentages to simulate fetal and maternal circulating cell-free DNA (ccfDNA) at various gestational weeks. The two ddPCR assays were then applied to determine the fetal genotype from 52 maternal plasma samples at different gestational ages. The diagnostic outcomes were confirmed for all the samples by DNA sequencing. In the case of mutations inherited from the mother or from both parents, a precise dosage of normal and mutated alleles was required to determine the fetal genotype. In particular, we identified two diagnostic ranges for allelic ratio values statistically distinct and not overlapping, allowing correct fetal genotype determinations for almost all the analyzed samples. In conclusion, we have developed a simple and sensitive diagnostic tool, based on ddPCR, for the NIPT of β^+^IVSI-110 and β^0^39 mutations paternally and, for the first time, maternally inherited, a tool, which may be applied to other single point mutations causing monogenic diseases.

## 1. Introduction

Non-invasive prenatal testing (NIPT) is based on the detection and characterization of circulating cell-free fetal DNA (ccffDNA) in maternal plasma [1], using a simple maternal peripheral blood sampling technique and avoiding risks associated with conventional invasive procedures, such as amniocentesis and chorionic villus sampling. 

ccffDNA comprises only a small portion (10–20%) of total circulating cell-free DNA (ccfDNA) in the last gestational weeks [2] and is detectable from the 4th week of gestation until delivery [3], increasing by 0.1% every week between the 10th and 21st gestational weeks and by almost 1% increment/week after the 21st week [4,5].

In addition, the amount of ccffDNA depends, besides the gestation period, on several other factors, such as maternal characteristics and body weight [5,6,7], aneuploidies [5] and twin pregnancies [8]. In any case, the very low amount of ccffDNA in maternal plasma is a critical point, requiring both specific and optimized techniques for its purification and highly sensitive detection approaches. 

NIPT has been applied to determine fetal sex [9,10], fetal rhesus D (RhD) genotyping [11,12], some pregnancy-associated conditions, including preeclampsia [13,14,15], aneuploidies [16,17] and the identification of paternally inherited monogenic disorders [16,18,19]. More recently, significant advancements have been reported, extending the potential applications to fetal whole-genome sequencing and maternally inherited mutations [20,21,22,23,24,25,26,27,28].

At present, commercial NIPT assays can detect only aneuploidies, fetal sex and small deletions or insertions, but not maternally inherited single point mutations [23,24,25]. 

In this context, innovative and sensitive approaches for NIPT of fetal single-gene point mutations are greatly needed. 

Recently, we have applied droplet digital PCR (ddPCR) technology to NIPT of Y chromosome at early gestational ages (prior to 7 weeks) for fetal sex determination. In particular, all maternal plasma samples were correctly determined for SRY gene detection using ddPCR even at very early gestational ages (4.5 weeks), achieving an unprecedented level of 100% accuracy [29]. 

The ddPCR technology is based on the partition of nucleic acid samples in thousands of nanoliter-sized droplets by water–oil emulsion droplet technology, reducing costs, preserving precious samples and detecting rare DNA target copies with high sensitivity [30,31].

For all these reasons, in this study, we decided to validate the ddPCR technology for NIPT of two single point mutations causing β thalassemia. 

β thalassemia is an autosomal recessive inherited blood disorder associated with the absence (β^0^) or reduction (β^+^) of adult hemoglobin, inducing severe anemia [32]. The therapeutic treatments based on blood transfusions and iron chelation are helpful but not curative. β thalassemia, relatively frequent in people of Mediterranean origin (such as Italy and Greece), is caused by more than 300 mutations of the β globin gene [32]. Patients inherit one mutated β globin allele from each parent, their parents being healthy carriers with just one mutated β globin gene. The two most frequent mutations in the Mediterranean area here investigated are β^+^IVSI-110 (a splicing site mutation) and β^0^39 (a nonsense codon mutation) [32,33].

In this work, we have optimized and developed two ddPCR-based NIPT assays for the above-mentioned mutations (maternally and/or paternally inherited) by fetal genotyping performed on 52 maternal plasma samples at different gestational ages (5th–39th gestational week). 

## 2. Materials and Methods

### 2.1. Collection of Samples

Blood samples from pregnant women were collected using test tubes containing EDTA anticoagulant after approval by the Ethical Committee (Prot. 67-2012) of University Hospital S. Anna, Ferrara (Italy) and the Thalassemia and Hemoglobinopathies Center, Laiko General Hospital, Athens (Greece). In all cases, informed consent was obtained and the experiments were conducted in agreement with the Declaration of Helsinki. A progressive number was assigned to each specimen to ensure the anonymity of the donor.

### 2.2. Preparation of Plasma

Plasma was prepared within 3 h from blood collection, according to the protocol previously described in the literature [29]. Briefly, after mixing tubes in a rotator for 5–10 min, samples were centrifuged at 1200× *g* for 10 min at 4 °C without brake. Plasma was then carefully collected and centrifuged again at 2400× *g* for 20 min at 4 °C in order to completely remove platelets and precipitates. The resulting supernatant was collected and stored at −80 °C in aliquots, so as not to thaw them more than once.

### 2.3. Extraction of Genomic DNA

Genomic DNA was extracted either by 0.5 mL of blood or by two buccal swabs using the QIAamp^®^ DNA Blood Mini Kit (Qiagen, Hilden, Germany) according to the manufacturer’s instructions. Two 60 μL DNA elutions were performed.

Collected DNA was checked by agarose gel electrophoresis, spectrophotometrically quantified and stored at −20 °C. UV spectrophotometric quantification was performed with a SmartSpec™ Plus spectrophotometer (Bio-Rad, Hercules, CA, USA). 

The enzymatic digestion of genomic DNA was carried out using SspI restriction enzyme (Thermo Fisher Scientific, Waltham, MA, USA) in order to simulate ccfDNA. It was chosen because it is able to recognize and cut two regions of the β globin gene, upstream of the promoter and inside the second intron, respectively, leaving the region around the mutation intact to avoid interference in primers and probe hybridization. Each reaction had a final volume of 20 μL, containing 50 ng of genomic DNA, 1 U of SspI enzyme and 1× buffer G (Thermo Fisher Scientific). The reaction was incubated at 37 °C for 16 h and finally the enzyme was inactivated at 65 °C for 20 min.

### 2.4. Extraction of Circulating Cell-Free DNA

As previously reported [29], ccfDNA was extracted from 2 mL of maternal plasma, not thawed more than once, using a QIAamp^®^ DSP Virus Spin Kit (Qiagen), according to the manufacturer’s instructions. DNA elution was performed in 60 μL of AVE buffer.

### 2.5. Synthetic Oligonucleotides

Synthetic oligonucleotides used as primers in polymerase chain reactions (PCR) and sequencing reactions were purchased from Sigma-Aldrich (St. Louis, MO, USA), while assays for ddPCR, containing differently labeled normal and mutated probes for each mutation under investigation, were purchased from Thermo Fisher Scientific and Bio-Rad. 

### 2.6. Sequencing Reaction

Sequencing reactions were performed using, as templates, β globin PCR products obtained from genomic DNA from healthy donors, healthy carriers, β thalassemia patients, future mothers or fathers or from babies. PCR amplification was carried out with the primer pairs BG1F (5′-GTGCCAGAAGAGCCAAGGACAGG-3′)–BG1R (5′-AGTTCTCAGGATCCACGTGCA-3′), BG2F (5′-GCCTGGCTCACCTGGACA-3′)–BG2R (5′-GTTGCCCAGGAGCTGTG-3′) and BG3F (5′-ACAATCCAGCTACCATTCTGCTTT-3′)–BG3R (5′-CACTGACCTCCCACATTCCCTTTT-3′). Each PCR was prepared in a final volume of 50 μL containing 1× ExTaq Buffer (Takara Bio, Nojihigashi, Kusatsu, Shiga, Japan), 12.5 μM dNTPs, 150 ng PCR primers, 1.25 U/reaction ExTaq DNA polymerase (Takara Bio) and 100 ng or 30 ng of human genomic DNA obtained from blood or buccal swabs, respectively. After a first denaturation step at 94 °C for 2 min, 35 amplification cycles were performed, including denaturation at 94 °C for 30 s, annealing at 1–2 degrees lower than the melting temperature of primers for 30 s and elongation at 72 °C for a time proportional to the product length. At the end, reactions were maintained at 72 °C for 10 min.

PCR products were analyzed by agarose gel electrophoresis before being purified from unincorporated PCR primers using the MicroClean reagent (Microzone Limited, Haywards Heath, West Sussex, UK).

Sequencing reactions were carried out according to Sanger’s method [34] with the same primers employed for PCR amplification. The reactions were performed in a final volume of 20 μL containing 40–90 ng of purified PCR template, 3.2 pmol of sequencing primer and 8 μL of Terminator Ready Reaction Mix of ABI PRISM^®^ BigDye™ Terminator Cycle Sequencing Ready Reaction Kit (Thermo Fisher Scientific). A total of 45 amplification cycles were performed as follows: denaturation, 96 °C, 10 s; annealing, 65 °C, 5 s; elongation, 65 °C, 3 min. After purification of the reaction products using Sephadex™ G-50 Superfine (GE Healthcare, Chicago, IL, USA), sequencing was finally performed by BMR Genomics (Padua, Italy), while the obtained sequence data were analyzed by Sequence Scanner, version 1.0 (Applied Biosystems, Thermo Fisher Scientific) software.

### 2.7. Droplet Digital™ PCR (ddPCR)

ddPCR was performed using the QX200™ Droplet Digital™ PCR system (Bio-Rad) and β^+^IVSI-110 (primers 5′-GGGTTTCTGATAGGCACTGACT-3′ and 5′-GCAGCCTAAGGGTGGGAAA-3′, Beta-110 N probe for not mutated sequence 5′-VIC^®^-CTCTGCCTATTGGTCTAT-NFQ-3′, Beta-110 M probe for mutated sequence 5′-FAM^TM^- TCTCTGCCTATTAGTCTAT-NFQ-3′; Thermo Fisher Scientific) or β^0^39 (ID dHsaMDS696192379, Bio-Rad) assays for detection of β^+^IVSI-110 and β^0^39 mutations, respectively. The reactions were prepared in a final volume of 22 μL containing 1× ddPCR^TM^ Supermix for Probes (no dUTP) (Bio-Rad), 1× β^+^IVSI-110 or 0.75× β^0^39 assay and 1 ng of genomic DNA, 10 ng of genomic DNA mixtures or 8 μL of ccffDNA template. 

The QX200^TM^ AutoDG^TM^ Droplet Generator (Bio-Rad) was then used to generate the emulsion droplets before amplification in an IQ^TM^5 Multicolor Real-Time PCR Detection System (Bio-Rad) under the following conditions: 10 min at 95 °C, 45 (for β^+^IVSI-110) or 40 (for β^0^39) cycles of a two-step thermal profile of 30 s at 94 °C and 1 min at the selected annealing and extension temperature, a final hold of 10 min at 98 °C, ramping rate 2 °C/s. The droplet fluorescence analysis was performed with a QX200^TM^ Droplet Reader (Bio-Rad) in combination with QuantaSoft 1.3.2.0 software (Bio-Rad) for data acquisition and calculation of the absolute concentration of target DNA (in copies/μL of reaction) using Poisson distribution analysis [30,31].

### 2.8. Statistical Analysis

After determining the numbers of positive droplets, negative droplets and double-positive droplets, to determine the disease status of a test sample, the ratio of the total copy number of the mutant allele to the total copy number of the wild-type allele (R_M:N_) and the z-score were calculated, as previously described by Tan et al. [35]. To be conservative, a 99% confidence bound, corresponding to a z-score cutoff of 2.58 standard deviations, was used to classify the samples’ genotypes. Numerical calculations were performed in Microsoft^®^ Excel^®^ software according to the equations and Poisson distribution models described in Tan et al. [35]. 

Statistical differences between groups were compared by means of the Student’s *t*-test, with a 99% confidence level selected. Statistical significance was assumed at *p* < 0.01. 

## 3. Results

### 3.1. Validation of β^+^IVSI-110 and β^0^39 Thalassemia ddPCR Assays by Genomic DNA Mixtures Mimicking Circulating Cell-Free Fetal DNA and Maternal DNA

After the optimization of experimental conditions for β^+^IVSI-110 (Figure S1,Supplementary Materials) and β^0^39 (Figure S2, Supplementary Materials) thalassemia ddPCR assays, both assays were validated for use in NIPT of β thalassemia.

Circulating cell-free DNA in maternal circulation is highly fragmented [36], 99% of fetus-derived DNA being shorter than 312 bp, whereas maternal cell-free DNA is longer than 300 bp with a median length of about 400–500 bp [37,38,39]. 

**Figure 1:**
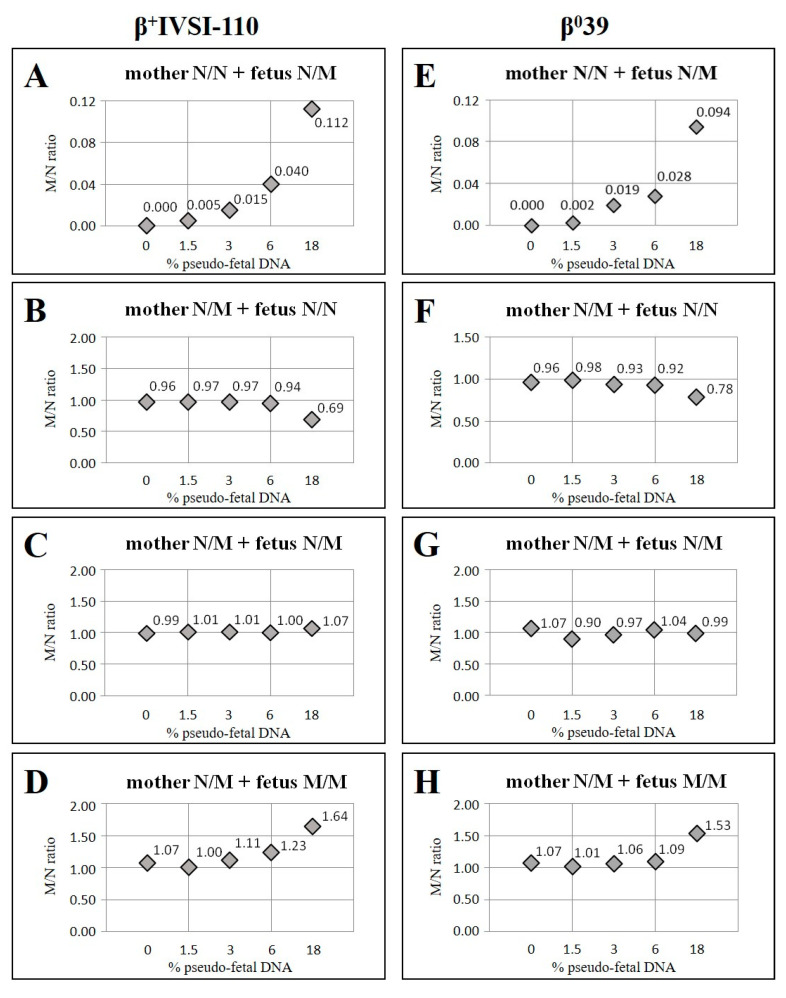
Graphical representation of ddPCR analysis of digested genomic DNA mixtures in order to validate β^+^IVSI-110 and β^0^39 assays. Digested genomic DNA mixtures of different genotypes were analyzed by ddPCR in order to simulate the maternal and fetal cell-free DNA of different genotypes with reference to β^+^IVSI-110 (**A**–**D**) or β^0^39 (**E**–**H**) mutations: N/N normal homozygous mother and N/M heterozygous fetus (**A**,**E**); N/M heterozygous mother and N/N homozygous normal (**B**,**F**) or N/M heterozygous (**C**,**G**) or M/M homozygous mutated (**D**,**H**) fetus. After β^+^IVSI-110 (**A**–**D**) or β^0^39 (**E**–**H**) ddPCR analysis, the M/N allelic ratios (indicated with diamond shapes) were obtained. For each graph, the percentage of analyzed pseudo-fetal DNA is reported.

Therefore, in order to simulate the fragmentation of ccfDNA, genomic DNA was digested using SspI restriction enzymes before the ddPCR experiment. In order to validate the β^+^IVSI-110 and β^0^39 thalassemia ddPCR assays, we tested them using mixtures of digested genomic DNAs of different genotypes (Figure 1) with the aim of simulating the actual targets of NIPT: circulating cell-free maternal and fetal DNAs. In the plasma of pregnant women, the percentage of ccffDNA is usually between 3% and 10–20% of the total ccfDNA [4]; therefore 0%, 1.5%, 3%, 6% and 18% were chosen as the percentages of pseudo-fetal DNA. For each β thalassemia mutation, four experiments were performed, simulating different maternal and fetal genotypes: normal homozygous mother (N/N) and heterozygous fetus (N/M) (Figure 1A,E), heterozygous mother (N/M) and normal homozygous (N/N) (Figure 1B,F), heterozygous (N/M) (Figure 1C,G) or mutated homozygous (M/M) fetus (Figure 1D,H). 

Both for β^+^IVSI-110 (Figure 1A–D) and β^0^39 (Figure 1E–H) mutations, when the pseudo-mother did not carry the mutation (N/N), only the normal sequences were amplified and the M/N ratio was equal to 0 (Figure 1A,E, 0% pseudo-fetal DNA); while, when the pseudo-fetus was heterozygous for the mutation, the mutated fetal sequences were also amplified, with the M/N ratio different from 0 but much lower than 1, considering that the amount of fetal DNA is lower than the amount of circulating cell-free maternal DNA (Figure 1A,E). Moreover, this M/N allelic ratio increased with increasing pseudo-fetal DNA percentages, as expected.

On the contrary, when the pseudo-mother was heterozygous (N/M), both the normal and the mutated alleles were amplified, and the M/N ratio was around 1 (Figure 1B–D,F–H, 0% pseudo-fetal DNA). In this situation, the digital relative mutation dosage (RMD) method described by Lun and colleagues [2] should be considered, based on dosages of the mutant and wild-type alleles of a disease-causing gene in maternal plasma, whose balance depends on the fetal genotype. Indeed, when the pseudo-fetus did not carry the mutation, as expected, a mild allelic imbalance was observed due to the fetal contribution, resulting in an M/N ratio decrease with the increase in the percentage of pseudo-fetal DNA (Figure 1B,F); while, if the pseudo-fetus was homozygous for the same maternal mutation, a mild allelic imbalance was obtained, with an enhancement of M/N ratio with the increasing percentage of pseudo-fetal DNA (Figure 1D,H). Finally, if the pseudo-fetus was heterozygous, like the mother, no imbalance occurred, resulting in an expected M/N ratio around 1, even when the percentage of pseudo-fetal DNA increased (Figure 1C,G). 

The obtained M/N allelic ratios (Obt) are reported in Table 1, together with the expected values (Exp). In order to evaluate how similar they were, the parameter (Obt/Exp) × 100 was calculated: the closer this value is to 100, the more similar the ratios are. Since for all samples the discrepancy was found to be lower than 15%, we can conclude that the M/N ratio is a good parameter to efficiently determine the allelic imbalance due to the pseudo-fetal DNA contribution. Therefore, considering the promising results, the M/N ratio could be applied and employed in a prenatal diagnostic view.

### 3.2. NIPT of β^+^IVSI-110 or β^0^39 Thalassemia Mutations Using ddPCR Assays

Fifty-two plasma samples collected from pregnant women at different gestational ages were analyzed using β^+^IVSI-110 or β^0^39 mutation ddPCR assays. For each sample, parental genotypes were determined by sequencing of the β globin gene after genomic DNA purification from blood or buccal swab. 

**Figure 2:**
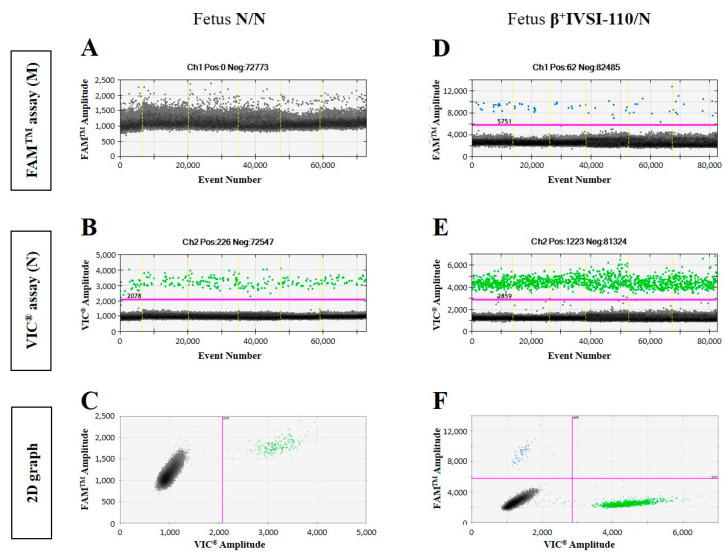
Representative examples of ddPCR graphs obtained by ccfDNAs from the plasma of normal homozygous N/N pregnant women with a partner who is a carrier of the β^+^IVSI-110 mutation (β^+^IVSI-110/N). ddPCR analysis outputs obtained by samples with N/N (sample #2, (**A**–**C**)) or β^+^IVSI-110/N (sample #8, (**D**–**F**)) fetuses are reported as representative results produced by different fetal genotypes. One-dimensional graphs relative to FAM^TM^ fluorescence corresponding to the mutated allele (**A**,**D**) and to VIC^®^ fluorescence corresponding to the normal allele (**B**,**E**) are reported, in addition to two-dimensional graphs showing both the fluorescence intensities (**C**,**F**). Positive events generated by mutated and normal alleles are shown in blue and green, respectively, while black dots indicate negative droplets. The threshold lines are colored fuchsia.

The determination of paternally inherited fetal mutations is the easiest case: if the mother is normal homozygous, the presence of positive events for the mutated allele is only due to the fetal contribution. Therefore, the 23 ccfDNA samples analyzed and the diagnostic outcomes obtained after ddPCR analysis are reported in Table 2. The sample was considered homozygous for the normal allele if no positive events for the mutated allele were detected and heterozygous for the specific mutation if more than three positive events for the mutated allele were detected. Figure 2 shows representative examples of ddPCR 1D and 2D graphs obtained by analysis of ccfDNAs from the plasma of normal homozygous N/N pregnant women carrying a N/N (Figure 2A–C) or β^+^IVSI-110/N (Figure 2D–F) fetus, respectively, whereas similar representative examples referred to the β^0^39 mutation are reported in Figure S3 (Supplementary Materials).

After the recovery of the newborn buccal swab and the extraction of genomic DNA, the diagnostic outcome was confirmed by DNA sequencing (Table 2). For all the samples analyzed, the diagnostic outcome was confirmed, also at a very early gestational age (fifth week).

In conclusion, for the first time, we have demonstrated that ddPCR technology can be used for NIPT of paternally inherited β^+^IVSI-110 and β^0^39 mutations at early gestational ages (prior to 9th week), confirming that ddPCR is a robust, sensitive, efficient and reliable technology [29].

The case of mutations inherited from the mother or both parents is more complex: a precise quantification of normal and mutated alleles in maternal plasma is required to evaluate whether there is a balanced or unbalanced M/N allelic ratio, according to the RMD approach described for the NIPT of monogenic diseases [2]. When the fetal genotype is identical to the mother’s (i.e., heterozygous), an allelic balance is expected; on the contrary, if the fetus is homozygous for the wild-type or the mutant allele, an allelic imbalance occurs.

Table 3 shows the 30 samples collected from pregnant women at different gestational ages (from the 7th to the 39th week) heterozygous for the β^+^IVSI-110 (on the left side) and β^0^39 (on the right side) mutations with a partner who is normal homozygous (in the upper part) or a carrier of the same maternal mutation (in the lower part).

The samples for which both the parents are carriers of the same mutation (from #20 to #24 for β^+^IVSI-110, #49 for β^0^39) are highly informative and interesting from a diagnostic point of view because they represent a real case in which the fetus could become a β thalassemia patient, e.g., sample #23.

In these cases, also, after recovery of the newborns’ buccal swabs and extraction of genomic DNA, the genotype of each sample was determined by DNA sequencing (Table 3).

**Figure 3:**
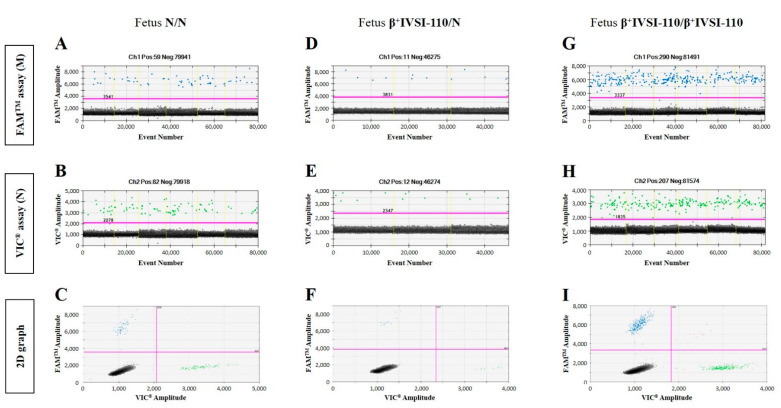
Representative examples of ddPCR graphs obtained by ccfDNAs from the plasma of heterozygous β^+^IVSI-110/N pregnant women with partners who are homozygous normal or carriers of the same mutation. ddPCR analysis outputs obtained by samples with N/N (sample #19, (**A**–**C**)), β^+^IVSI-110/N (sample #24, (**D**–**F**)) or β^+^IVSI-110/β^+^IVSI-110 (sample #23, (**G**–**I**)) fetuses are reported as representative results achieved by different fetal genotypes. One-dimensional graphs, relative to FAM^TM^ fluorescence corresponding to the mutated allele (**A**,**D**,**G**) and to VIC^®^ fluorescence corresponding to the normal allele (**B**,**E**,**H**), are reported, in addition to 2D graphs showing both of the fluorescence intensities (**C**,**F**,**I**). Positive events generated by mutated and normal alleles are shown in blue and green, respectively, while black dots indicate negative droplets. The threshold lines are colored fuchsia.

Figure 3 shows representative examples of ddPCR 1D and 2D graphs obtained by analysis of ccfDNAs from the plasma of heterozygous β^+^IVSI-110/N pregnant women carrying a N/N (Figure 3A–C), β^+^IVSI-110/N (Figure 3D–F) or β^+^IVSI-110/β^+^IVSI-110 (Figure 3G–I) fetus, respectively. Similar representative examples referred to the β^0^39 mutation are shown in Figure S4 (Supplementary Materials), where 1D and 2D graphs obtained by analysis of ccfDNAs from the plasma of heterozygous β^0^39/N pregnant women carrying a N/N or β^0^39/N fetus, respectively, are reported. According to the RMD method [2], the concentration of both alleles was measured (copies/µL) and the M/N allelic ratio calculated for each sample (Table S1, Supplementary Materials).

**Figure 4:**
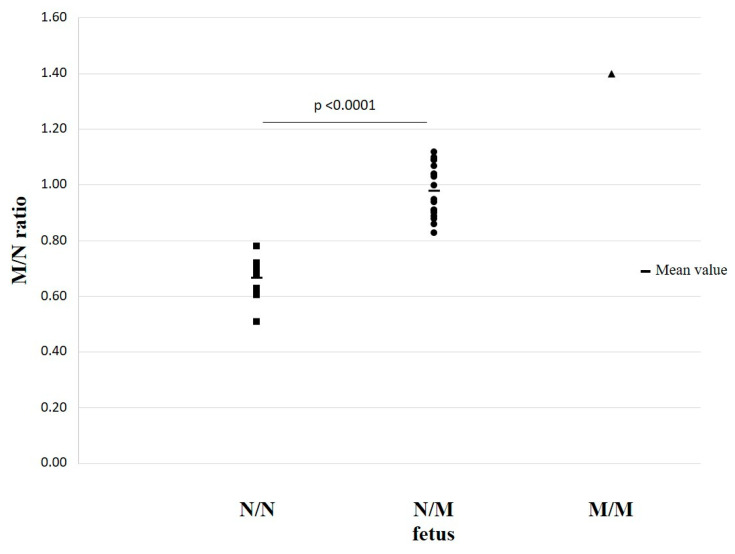
M/N allelic ratio distribution through ddPCR to detect β thalassemia fetal genotypes from ccfDNAs of heterozygous pregnant women. All of the 30 ccfDNA samples analyzed, obtained from pregnant women heterozygous for the β^+^IVSI-110 or β^0^39 mutation, are displayed. Three distinct groups are identified: samples for which the fetus is not a carrier of the mutation (N/N, squares), samples for which the fetus is a carrier of the β^+^IVSI-110 or β^0^39 mutation (N/M, dots) and a sample for which the fetus is mutated homozygous (M/M, triangle). The mean value of each distribution and the statistical significance of the analysis are also indicated. The statistical difference between groups was evaluated by the Student’s *t*-test, with a 99% confidence level selected.

The distribution of M/N ratio values obtained from these samples is displayed in Figure 4, where each indicator represents a sample. Despite some variability in the calculated values, two distinct and not overlapping value populations were identified according to the different fetal genotypes: samples where the fetus was not a carrier of any mutation (N/N, square indicators) and samples where the fetus was heterozygous for the β^+^IVSI-110 or β^0^39 mutation (N/M, dot indicators). The relevant statistical analyses confirmed that the differences among the value populations obtained from different fetal genotypes were statistically significant.

Indeed, as expected, the samples from women carrying heterozygous fetuses showed an M/N ratio around 1. On the contrary, the samples from women carrying normal fetuses, where the allelic imbalance is due to an under-representation of the mutant allele compared to the normal one [2], showed an M/N ratio lower than 1 and so lower than the M/N ratios of the previous samples. Therefore, two diagnostic ranges based on M/N ratio values were identified: the first one, from 0.51 to 0.78 (mean value: 0.67 ± 0.07), related to normal fetuses, and the second, from 0.83 to 1.12 (mean value: 0.98 ± 0.09), related to heterozygous fetuses. The identified ranges are distinct, demonstrating the suitability of the developed ddPCR-based assays to correctly discriminate the different allelic fetus conditions for the β thalassemia mutations under investigation.

Finally, as regards the mutated homozygous fetal genotype, we found only one sample (#23) which gave an M/N ratio higher than 1 (1.40), as expected, but without the chance to obtain a statistical analysis, as for the two other genotypes.

We further investigated the confidence level of the results by calculating the z-scores (Table 3). An independent training set of 21 samples was used to define the parameters required for z-scoring [35]. In these samples the mean and standard deviation of the N/N samples were 0.82 and 0.18, respectively, whereas M/N samples had a mean equal to 1.03 with a standard deviation of 0.12.

The z-score classification was able to correctly assign 29 cases over 30. There was an incorrect classification for sample #45, showing the highest M/N ratio among the normal fetuses, and which the z-score classified as heterozygous (Table 3), most probably because the sample falls in the point of intersection of the upper bound of N/N samples and the lower bound of the M/N genotypes.

Table 3 summarizes the obtained results: for each sample, the single M/N ratio, the z-score, the formulated diagnosis using ddPCR, the fetal genotype determined by DNA sequencing and the diagnosis outcome are indicated. For 29 out of 30 of the analyzed samples, the diagnostic outcome was confirmed, also at early gestational weeks (until the 7th week).

## 4. Discussion

Recently, NIPT based on advanced technologies to investigate ccffDNA has allowed the determination of fetal sex, fetal rhesus D (RhD) genotyping, aneuploidies, micro-deletions and the detection of only paternally inherited monogenic disorders [40]. An example of novel approaches (in this case also based on novel instrumental devices) is droplet digital PCR (ddPCR), an innovative and sensitive strategy useful for precise and absolute quantification of nucleic acids, indicated for allelic variant investigation and target gene identification using very low amounts of the starting sample to be analyzed. The possible use of ddPCR for the NIPT of monogenic diseases has already been described [20,28,41].

In this article, we demonstrated for the first time that ddPCR technology can be used for the NIPT of β^+^IVSI-110 and β^0^39 mutations, both maternally and paternally inherited. These data confirm that the ddPCR is a robust, sensitive, efficient and reliable technology to easily detect single point mutations. The second consideration supporting the novelty of our approach is that we have been able to perform NIPT using seventh week sampling. This remarkable timing has not been described so far for ddPCR-based NIPT. Actually, Sawakwongpra and colleagues described a ddPCR-based approach for the NIPT of β thalassemia, but they have been able to perform NIPT only from the 17th gestational week [28]. In addition, this method is independent of the fetal fraction, making it cost- and time-effective.

With respect to the aforementioned points, we would like to underline that the NIPT of maternally inherited mutations has been recently reported employing approaches other than ddPCR [20,42]. For instance, Yang et al. [42] described a cell-free DNA barcode-enabled single-molecule test (cfBEST) to detect low-abundance mutations in cfDNA for thalassemia. While cfBEST is comparable to ddPCR in terms of sensitivity and efficiency, it requires multiple PCR steps [42]. Another example has been reported by Perlado et al. [20], who applied ddPCR for fetal allele detection independently of parental origin using single nucleotide polymorphisms (SNPs). Unlike our approach, these authors were able to apply this method with up to 11-week samples and not for earlier gestational ages.

In conclusion, in this study we report for the first time a simple, fast and sensitive NIPT approach for the identification of maternally inherited β^+^IVSI-110 and β^0^39 thalassemia mutations, suitable for application at very early gestational ages, such as the seventh gestation week.

## Figures and Tables

**Table 1:** M/N allelic ratio values obtained from ddPCR analysis of digested genomic DNA mixtures in order to validate β^+^IVSI-110 and β^0^39 assays. For each simulated maternal/fetal combination, the expected M/N allelic ratio, the obtained one and the calculated obtained (Obt)/expected (Exp) parameter (as percentages) are reported, in addition to the percentage of analyzed pseudo-fetal DNA.

Mother β^+^IVSI-110/N with Fetus N/N				Mother β^0^39/N with Fetus N/N			
Pseudo-Fetal DNA (%)	Expected M/N Ratio	Obtained M/N Ratio	Obt/Exp × 100	Pseudo-Fetal DNA (%)	Expected M/N Ratio	Obtained M/N Ratio	Obt/Exp × 100
0	1.00	0.96	96	0	1.00	0.96	96
1.5	0.97	0.97	100	1.5	0.97	0.98	101
3	0.94	0.97	103	3	0.94	0.93	99
6	0.89	0.94	106	6	0.89	0.92	104
18	0.69	0.69	100	18	0.69	0.78	112
**Mother β^+^IVSI-110/N-with Fetus β^+^IVSI-110/β^+^IVSI 110**				**Mother β^0^39/N with Fetus β^0^39/β^0^39**			
**Pseudo-Fetal DNA (%)**	**Expected M/N Ratio**	**Obtained M/N Ratio**	**Obt/Exp × 100**	**Pseudo-Fetal DNA (%)**	**Expected M/N Ratio**	**Obtained M/N Ratio**	**Obt/Exp × 100**
0	1.00	1.07	107	0	1.00	1.07	107
1.5	1.03	1.00	97	1.5	1.03	1.01	98
3	1.06	1.11	105	3	1.06	1.06	100
6	1.13	1.23	109	6	1.13	1.09	97
18	1.44	1.64	114	18	1.44	1.53	106
**Mother β^+^IVSI-110/N with Fetus β^+^IVSI-110/N**				**Mother β^0^39/N with Fetus β^0^39/N**			
**Pseudo-Fetal DNA (%)**	**Expected M/N Ratio**	**Obtained M/N Ratio**	**Obt/Exp × 100**	**Pseudo-Fetal DNA (%)**	**Expected M/N Ratio**	**Obtained M/N Ratio**	**Obt/Exp × 100**
0	1.00	0.99	99	0	1.00	1.07	107
1.5	1.00	1.01	101	1.5	1.00	0.90	90
3	1.00	1.01	101	3	1.00	0.97	97
6	1.00	1.00	100	6	1.00	1.04	104
18	1.00	1.07	107	18	1.00	0.99	99

**Table 2:** Lists of the 23 samples collected from normal homozygous pregnant women with a partner who is a carrier of the β^+^IVSI-110 (on the left side) or β^0^39 (on the right side) mutation. For each sample, the assigned number and the gestational age are indicated. The samples are heterogeneous in terms of gestational weeks (from 39 to 5). **√**, confirmed.

Pregnant Women N/N with Partner β^+^IVSI-110/N						Pregnant Women N/N with Partner β^0^39/N					
Sample	Gestational Weeks	Positive Events for M Allele (no.)	Formulated Diagnosis	Fetal Genotype	Diagnosis Outcome	Sample	Gestational Weeks	Positive Events for M Allele (no.)	Formulated Diagnosis	Fetal Genotype	Diagnosis Outcome
1	37	0	N/N	N/N	**√**	25	39	196	β^0^39/N	β^0^39/N	**√**
2	18	0	N/N	N/N	**√**	26	35	123	β^0^39/N	β^0^39/N	**√**
3	16	91	β^+^IVSI-110/N	β^+^IVSI-110/N	**√**	27	33	39	β^0^39/N	β^0^39/N	**√**
4	15	43	β^+^IVSI-110/N	β^+^IVSI-110/N	**√**	28	29	19	β^0^39/N	β^0^39/N	**√**
5	12	1	N/N	β^0^IVSII-1/N	**√**	29	28	78	β^0^39/N	β^0^39/N	**√**
6	12	12	β^+^IVSI-110/N	β^+^IVSI-110/β^0^39	**√**	30	26	33	β^0^39/N	β^0^39/N	**√**
7A	10	178	β^+^IVSI-110/N	β^+^IVSI-110/N	**√**	31	25	21	β^0^39/N	β^0^39/N	**√**
8	9	62	β^+^IVSI-110/N	β^+^IVSI-110/N	**√**	32	24	2	N/N	N/N	**√**
7B	5	3	β^+^IVSI-110/N	β^+^IVSI-110/N	**√**	33	24	8	β^0^39/N	β^0^39/N	**√**
						34	21	39	β^0^39/N	β^0^39/N	**√**
						35	14	50	β^0^39/N	β^0^39/N	**√**
						36	13	4	β^0^39/N	β^0^39/N	**√**
						37	7	0	N/N	N/N	**√**
						38	5	26	β^0^39/N	β^0^39/N	**√**

**Table 3:** Fetal genotype determination by ddPCR of ccfDNAs from the plasma of heterozygous pregnant women for β^+^IVSI-110 or β^0^39 mutations with partners who are homozygous normal or carriers of the same mutation. The table shows, for each sample from pregnant woman with heterozygous genotypes for β^+^IVSI-110 (β^+^IVSI-110/N) or β^0^39 (β^0^39/N) whose partner is normal homozygous (N/N) or heterozygous (β^+^IVSI-110/N; β^0^39/N) for the same mutation, the assigned number, the gestational age, the M/N allelic ratio, the calculated z-score value, the formulated diagnosis resulting from ddPCR assays, the actual fetal genotype, determined by DNA sequencing, and the diagnosis outcome. Sample #6 *: β^0^39/N maternal genotype and β^+^IVSI-110/N paternal genotype. **√**, confirmed.

Pregnant Women β^+^IVSI-110/N with Partner N/N						Pregnant Women β^0^39/N with Partner N/N					
# Sample	Gestational Weeks	M/N Ratio	z-Score	Formulated Diagnosis	Fetal Genotype (Diagnosis Outcome)	# Sample	Gestational Weeks	M/N Ratio	z-Score	Formulated Diagnosis	Fetal Genotype (Diagnosis Outcome)
9	38	0.61	−3.46	N/N	N/N (**√**)	39	39	1.09	0.52	β^0^39/N	β^0^39/N (**√**)
10	33	0.63	−3.37	N/N	N/N(**√**)	40	36	0.89	−1.19	β^0^39/N	β^0^39/N (**√**)
11	30	0.86	−1.41	β^+^IVSI-110/N	β^+^IVSI-110/N (**√**)	41	29	0.71	−2.66	N/N	N/N (**√**)
12	30	0.83	−1.64	β^+^IVSI-110/N	β^+^IVSI-110/N (**√**)	42A	26	1.00	−0.25	β^0^39/N	β^0^39/N (**√**)
13	14	0.91	−1.02	β^+^IVSI-110/N	β^+^IVSI-110/N (**√**)	43	26	0.51	−4.36	N/N	N/N (**√**)
14	12	1.03	0.00	β^+^IVSI-110/N	β^+^IVSI-110/N (**√**)	42B	22	1.09	0.49	β^0^39/N	β^0^39/N (**√**)
15	12	0.95	−0.67	β^+^IVSI-110/N	β^+^IVSI-110/N (**√**)	44	20	0.68	−2.93	N/N	N/N (**√**)
16	12	0.91	−1.00	β^+^IVSI-110/N	β^+^IVSI-110/N (**√**)	45	18	0.78	−2.06	β^0^39/N	N/N
17	11	0.91	−1.00	β^+^IVSI-110/N	β^+^IVSI-110/N (**√**)	46	14	0.63	−3.31	N/N	N/N (**√**)
18	10	1.04	0.09	β^+^IVSI-110/N	β^+^IVSI-110/N (**√**)	42C	13	1.12	0.75	β^0^39/N	β^0^39/N (**√**)
19	9	0.72	−2.59	N/N	N/N (**√**)	6 *	12	1.07	0.33	β^0^39/N	β^0^39/β^+^IVSI-110 (**√**)
**Pregnant Women β^+^IVSI-110/N with Partner β^+^IVSI-110/N**						47	9	0.88	−1.28	β^0^39/N	β^0^39/N (**√**)
**# Sample**	**Gestational Weeks**	**M/N Ratio**	**z-Score**	**Formulated Diagnosis**	**Fetal Genotype (Diagnosis Outcome)**	48	7	1.10	0.58	β^0^39/N	β^0^39/N (**√**)
						**Pregnant Women β^0^39/N with Partner β^0^39/N**					
						**# Sample**	**Gestational Weeks**	**M/N** **Ratio**	**z-Score**	**Formulated Diagnosis**	**Fetal Genotype (Diagnosis Outcome)**
20	12	0.94	−0.73	β^+^IVSI-110/N	β^+^IVSI-110/N (**√**)						
21	10	0.70	−2.71	N/N	N/N (**√**)						
22	8	1.04	0.09	β^+^IVSI-110/N	β^+^IVSI-110/N (**√**)	49	32	0.69	−2.82	N/N	N/N (**√**)
23	7	1.40	3.09	β^+^IVSI-110/β^+^IVSI-110	β^+^IVSI-110/β^+^IVSI-110 (**√**)						
24	7	0.90	−0.94	β^+^IVSI-110/N	β^+^IVSI-110/N (**√**)						

## Data Availability

All the data will be available upon request to the corresponding author.

## Supplementary Materials

The following supporting information can be downloaded at: https://www.mdpi.com/article/10.3390/ijms23052819/s1.
