# Supplementary material for: Droplet Digital PCR for Non-Invasive Prenatal Detection of Fetal Single-Gene Point Mutations in Maternal Plasma
Source: Int J Mol Sci. 2022 Mar 4;23(5):2819. doi: 10.3390/ijms23052819 (PMC8911123; doi:10.3390/ijms23052819)
Supplement: Supplementary file 1 [file ijms-23-02819-s001.zip › ijms-1594076-supplementary.pdf]

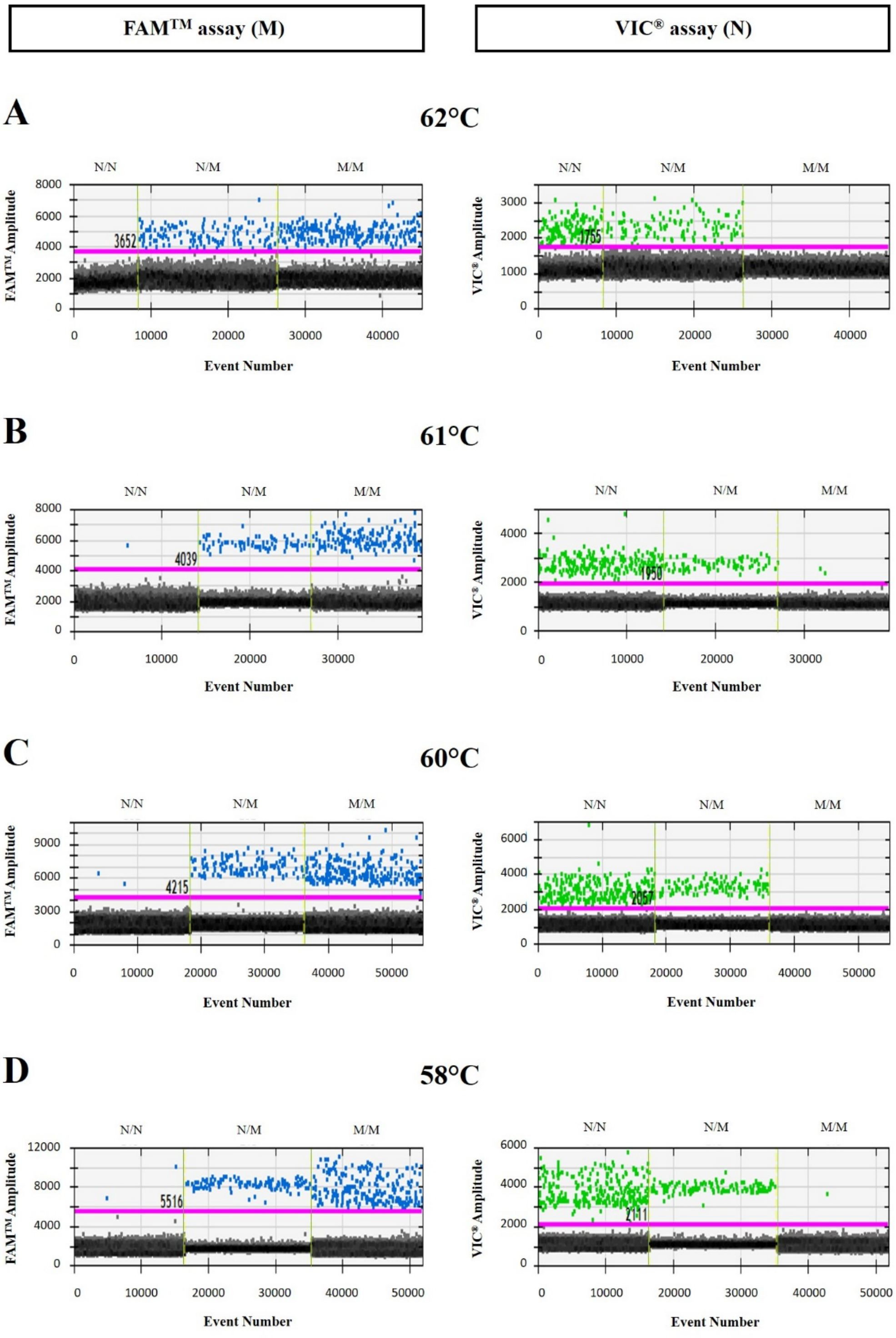

Figure S1. ddPCR graphs for not digested genomic DNA samples of different genotypes, for β^+IVSI-110 mutation, in order to set-up the assay annealing temperature. With the aim to set-up the ddPCR experimental conditions, normal homozygous (N/N), heterozygous (N/M) and mutated homozygous (M/M) for the β^+IVSI-110 mutation samples were analyzed in ddPCR, for mutated (in blue) and normal (in green) allele targets, at different annealing temperatures: 62°C (A), 61°C (B), 60°C (C) and 58°C (D). The graphs on the left side correlate the FAM™ fluorescence intensity, corresponding to the mutated allele (blue dots), to the number of events; the graphs on the right side report the VIC® fluorescence intensity, relative to the normal allele (green dots), to the number of events. Black dots indicate negative droplets (no amplification events). The threshold lines are colored in fuchsia.

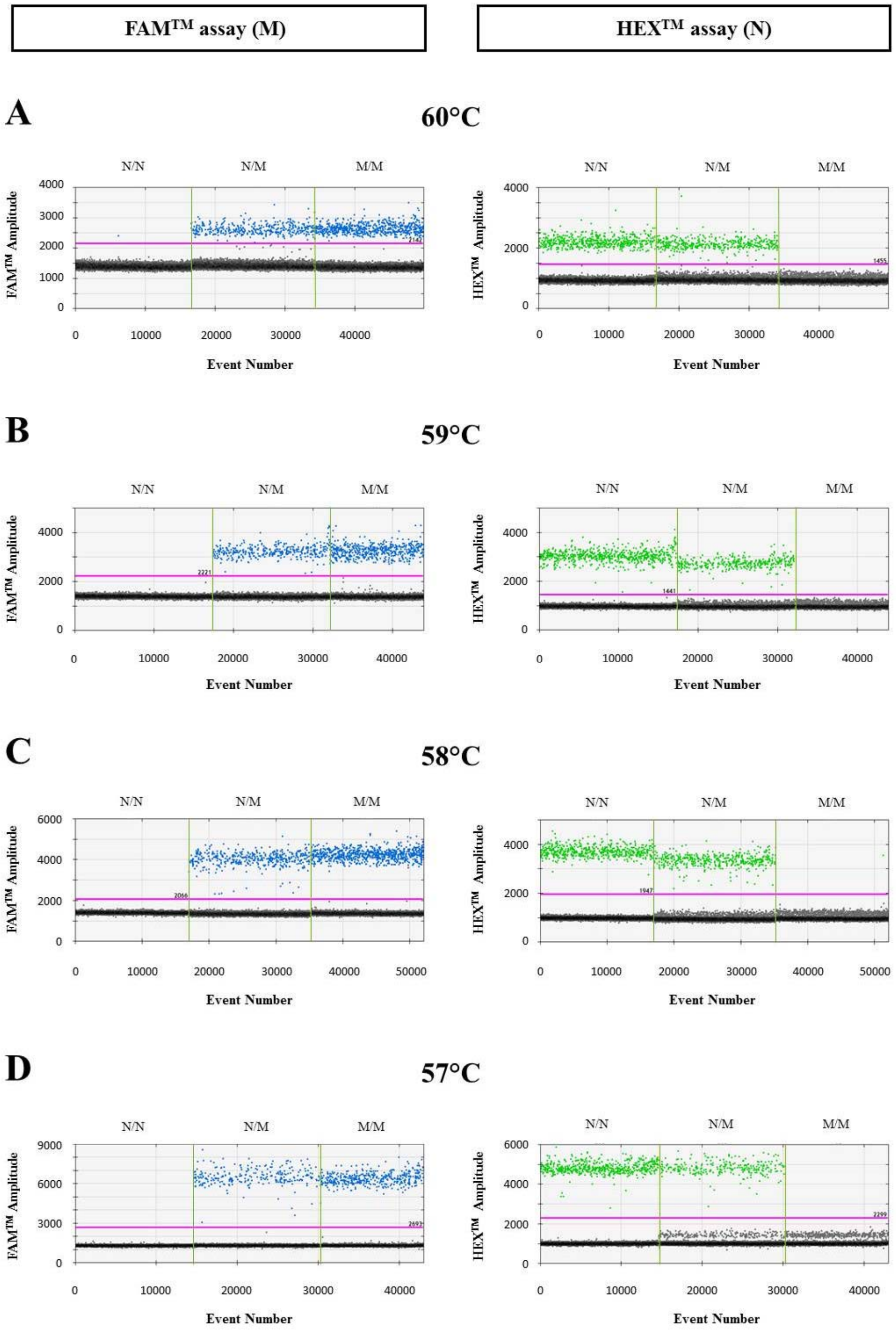

Figure S2. ddPCR graphs for not digested genomic DNA samples of different genotypes, for $\beta^0$39 mutation, in order to set-up the assay annealing temperature. With the aim to set-up the ddPCR experimental conditions, normal homozygous (N/N), heterozygous (N/M) and mutated homozygous (M/M) for the $\beta^0$39 mutation samples were analyzed in ddPCR, for mutated (in blue) and normal (in green) allele targets, at different annealing temperatures: 60°C (A), 59°C (B), 58°C (C) and 57°C (D). The graphs on the left side correlate the FAM™ fluorescence intensity, corresponding to the mutated allele (blue dots), to the number of events; the graphs on the right side report the HEX™ fluorescence intensity, relative to the normal allele (green dots), to the number of events. Black dots indicate negative droplets (no amplification events). The threshold lines are colored in fuchsia.

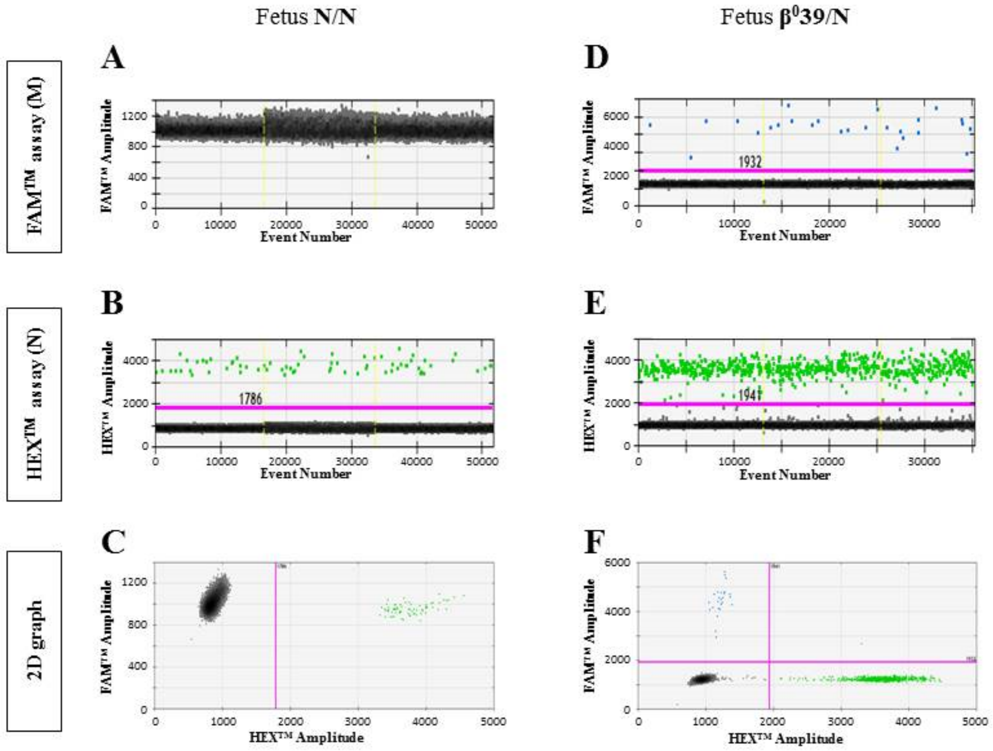

Figure S3. Representative examples of ddPCR graphs obtained by ccfDNAs from plasma of normal homozygous N/N pregnant women with partner carrier of $\beta^0$39 mutation ($\beta^0$39/N). ddPCR analysis outputs obtained by samples with N/N (sample # 37, A-C) or $\beta^0$39/N (sample # 38, D-F) fetus are reported as representative results produced by different fetal genotypes. 1D graphs, relative to FAM^{TM} fluorescence corresponding to the mutated allele (A,D) and to HEX^{TM} fluorescence corresponding to the normal allele (B,E) are reported, in addition to 2D graphs showing both the fluorescence intensities (C,F). Positive events generated by mutated and normal alleles are shown in blue and green, respectively, while black dots indicate negative droplets. The threshold lines are colored in fuchsia.

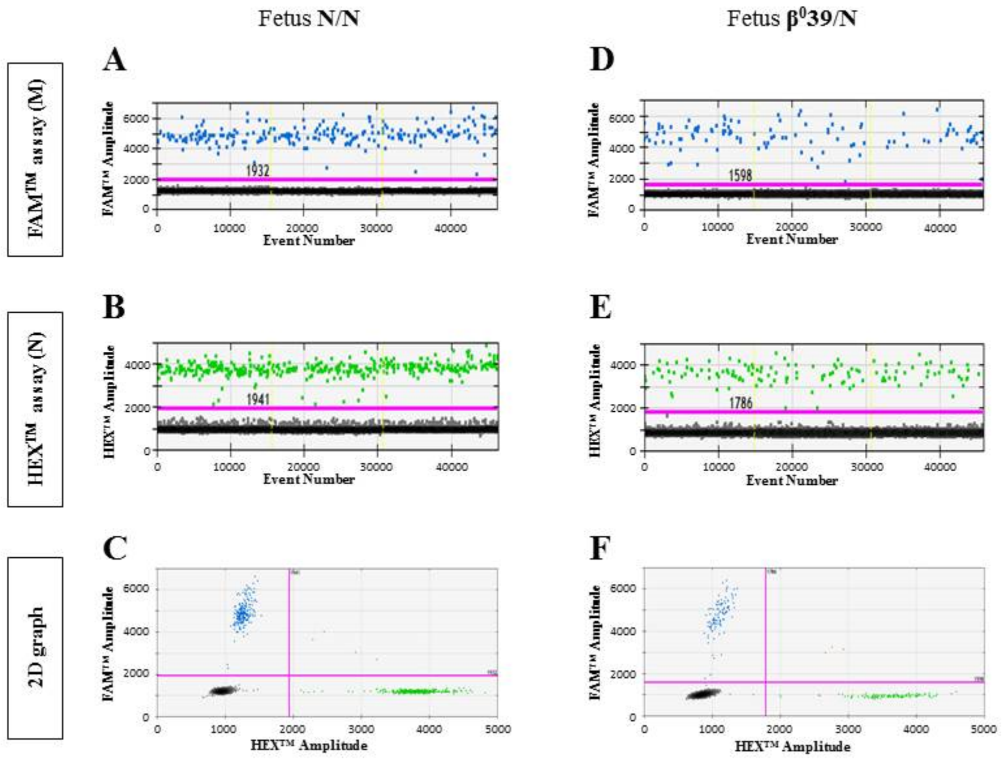

Figure S4. Representative examples of ddPCR graphs obtained by ccfDNAs from plasma of heterozygous $\beta^0$39/N pregnant women with partners homozygous normal or carrier of the same mutation. ddPCR analysis outputs obtained by samples with N/N (sample # 46, A-C) or $\beta^0$39/N (sample # 47, D-F) fetus are reported as representative results produced by different fetal genotypes. 1D graphs, relative to FAM^{TM} fluorescence corresponding to the mutated allele (A,D) and to HEX^{TM} fluorescence corresponding to the normal allele (B,E) are reported, in addition to 2D graphs showing both the fluorescence intensities (C,F). Positive events generated by mutated and normal alleles are shown in blue and green, respectively, while black dots indicate negative droplets. The threshold lines are colored in fuchsia.

Pregnant women β^{+}IVSI-110/N with partner N/N					
# sample	Positive events (no.)		Concentration (copies/µL)		M/N ratio
	M allele	N allele	M allele	N allele	
9	2138	3478	55.60	91.80	0.61
10	117	376	1.83	2.92	0.63
11	93	108	1.72	2.00	0.86
12	1224	1468	44.80	53.90	0.83
13	88	97	1.05	1.16	0.91
14	34	33	0.43	0.42	1.03
15	76	80	1.43	1.50	0.95
16	91	100	2.36	2.60	0.91
17	81	89	1.22	1.34	0.91
18	76	73	1.21	1.16	1.04
19	59	82	0.87	1.21	0.72

Pregnant women β^{+}IVSI-110/N with partner β^{+}IVSI-110/N					
# sample	Positive events (no.)		Concentration (copies/µL)		M/N ratio
	M allele	N allele	M allele	N allele	
20	66	70	1.36	1.44	0.94
21	110	156	1.72	2.43	0.70
22	203	195	2.88	2.76	1.04
23	290	207	4.18	2.98	1.40
24	11	12	0.28	0.31	0.90

Pregnant women β^{0}39/N with partner N/N					
# sample	Positive events (no.)		Concentration (copies/µL)		M/N ratio
	M allele	N allele	M allele	N allele	
39	83	76	6.20	5.60	1.09
40	118	133	10.60	12.00	0.89
41	76	107	7.70	10.90	0.71
42A	64	64	2.90	2.90	1.00
43	38	75	3.00	5.90	0.51
42B	123	113	5.60	5.10	1.09
44	368	542	18.80	27.70	0.68
45	195	249	8.20	10.50	0.78
46	243	384	6.20	9.80	0.63
42C	121	108	5.30	4.80	1.12
6	184	172	4.40	4.20	1.07
47	113	129	2.90	3.30	0.88
48	33	30	0.75	0.68	1.10

Pregnant women β^{0}39/N with partner β^{0}39/N					
# sample	Positive events (no.)		Concentration (copies/µL)		M/N ratio
	M allele	N allele	M allele	N allele	
49	224	324	8.10	11.70	0.69

Table S1. Results obtained after ddPCR analysis of ccfDNAs from plasma of heterozygous pregnant women for β^{+}IVSI-110 or β^{0}39 mutation with partners homozygous normal or carrier of the same mutation. For each sample from pregnant woman with heterozygous genotype, for β^{+}IVSI-110 (β^{+}IVSI-110/N) or β^{0}39 (β^{0}39/N), whose partner is normal homozygous (N/N) or heterozygous (β^{+}IVSI-110/N; β^{0}39/N) for the same mutation, the table shows the number of positive events obtained for mutated (M) and not mutated (N) alleles, the resulting concentration of both alleles (in copies/µL) and the calculated M/N allelic ratio.
